# Noise Suppression and Surplus Synchrony by Coincidence Detection

**DOI:** 10.1371/journal.pcbi.1002904

**Published:** 2013-04-04

**Authors:** Matthias Schultze-Kraft, Markus Diesmann, Sonja Grün, Moritz Helias

**Affiliations:** 1 Machine Learning Group, Berlin Institute of Technology, Berlin, Germany; 2 Bernstein Focus: Neurotechnology, Berlin, Germany; 3 Bernstein Center for Computational Neuroscience, Berlin, Germany; 4 Institute of Neuroscience and Medicine (INM-6), Computational and Systems Neuroscience, Jülich Research Centre, Jülich, Germany; 5 Medical Faculty, RWTH Aachen University, Aachen, Germany; 6 RIKEN Brain Science Institute, Wako City, Japan; 7 Theoretical Systems Neurobiology, RWTH Aachen University, Aachen, Germany; École Normale Supérieure, College de France, CNRS, France

## Abstract

The functional significance of correlations between action potentials of neurons is still a matter of vivid debate. In particular, it is presently unclear how much synchrony is caused by afferent synchronized events and how much is intrinsic due to the connectivity structure of cortex. The available analytical approaches based on the diffusion approximation do not allow to model spike synchrony, preventing a thorough analysis. Here we theoretically investigate to what extent common synaptic afferents and synchronized inputs each contribute to correlated spiking on a fine temporal scale between pairs of neurons. We employ direct simulation and extend earlier analytical methods based on the diffusion approximation to pulse-coupling, allowing us to introduce precisely timed correlations in the spiking activity of the synaptic afferents. We investigate the transmission of correlated synaptic input currents by pairs of integrate-and-fire model neurons, so that the same input covariance can be realized by common inputs or by spiking synchrony. We identify two distinct regimes: In the limit of low correlation linear perturbation theory accurately determines the correlation transmission coefficient, which is typically smaller than unity, but increases sensitively even for weakly synchronous inputs. In the limit of high input correlation, in the presence of synchrony, a qualitatively new picture arises. As the non-linear neuronal response becomes dominant, the output correlation becomes higher than the total correlation in the input. This transmission coefficient larger unity is a direct consequence of non-linear neural processing in the presence of noise, elucidating how synchrony-coded signals benefit from these generic properties present in cortical networks.

## Author Summary

Whether spike timing conveys information in cortical networks or whether the firing rate alone is sufficient is a matter of controversial debate, touching the fundamental question of how the brain processes, stores, and conveys information. If the firing rate alone is the decisive signal used in the brain, correlations between action potentials are just an epiphenomenon of cortical connectivity, where pairs of neurons share a considerable fraction of common afferents. Due to membrane leakage, small synaptic amplitudes and the non-linear threshold, nerve cells exhibit lossy transmission of correlation originating from shared synaptic inputs. However, the membrane potential of cortical neurons often displays non-Gaussian fluctuations, caused by synchronized synaptic inputs. Moreover, synchronously active neurons have been found to reflect behavior in primates. In this work we therefore contrast the transmission of correlation due to shared afferents and due to synchronously arriving synaptic impulses for leaky neuron models. We not only find that neurons are highly sensitive to synchronous afferents, but that they can suppress noise on signals transmitted by synchrony, a computational advantage over rate signals.

## Introduction

Simultaneously recording the activity of multiple neurons provides a unique tool to observe the activity in the brain. The immediately arising question of the meaning of the observed correlated activity between different cells [1], [2] is tightly linked to the problem how information is represented and processed by the brain. This problem is matter of an ongoing debate [3] and has lead to two opposing views. In one view, the high variability of the neuronal response [4] to presented stimuli and the sensitivity of network activity to the exact timing of spikes [5] suggests that the slowly varying rate of action potentials carries the information in the cortex. A downstream neuron can read out the information by pooling a sufficient number of merely independent stochastic source signals. Correlations between neurons may either decrease the signal-to-noise ratio [6] or enhance the information [7] in such population signals, depending on the readout mechanism. Correlations are an unavoidable consequence of cortical connectivity where pairs of neurons share a considerable amount of common synaptic afferents [8]. Recent works have reported very low average correlations in cortical networks on long time scales [9], explainable by an active mechanism of decorrelation [10], [11], [12]. On top of these correlations inherent to cortex due to its connectivity, a common and slowly varying stimulus can evoke correlations on a long time scale.

In the other view, on the contrary, theoretical considerations [13], [14], [15], [16] argue for the benefit of precisely timed action potentials to convey and process information by binding elementary representations into larger percepts. Indeed, in frontal cortex of macaque, correlated firing has been observed to be modulated in response to behavioral events, independent of the neurons' firing rate [17]. On a fine temporal scale, synchrony of action potentials [18], [19], [20] has been found to dynamically change in time in relation to behavior in primary visual cortex [21] and in motor cortex [17], [22]. The observation that nearby neurons exclusively show positive correlations suggests common synaptic afferents to be involved in the modulation of correlations [23]. In this view, the measure of interest are correlations on a short temporal scale, often referred to as synchrony.

The role of correlations entails the question whether cortical neurons operate as integrators or as coincidence detectors [18], [24]. Recent studies have shown that single neurons may operate in both regimes [25]. If the firing rate is the decisive signal, integrator properties become important, as neural firing is driven by the mean input. As activity is modulated by the slowly varying signal, correlations extend to long time scales due to co-modulation of the rate. Integrators are thus tailored to the processing of rate coded signals and they transmit temporal patterns only unreliably. Coincidence detectors preferentially fire due to synchronously arriving input. The subthreshold membrane potential fluctuations reflect the statistics of the summed synaptic input [26], which can be used to identify temporally precise repetition of network activity [27]. A direct probe for the existence of synchronous activity are the resulting strong deflections due to synchronous arrival of synaptic impulses. Such non-Gaussian fluctuations have indeed been observed in auditory cortex in vivo [28] and in the barrel cortex of behaving mice [29]. In this regime, coincidence detector properties become crucial. Coincidence detectors are additionally sensitive to stimulus variance [25], [30] and correlations between pairs of neurons in this regime arise from precisely timed firing. This type of correlation is unaffected by firing rate, can encode stimulus properties independently and moreover arises on short time scales [25].

**Figure 1:**
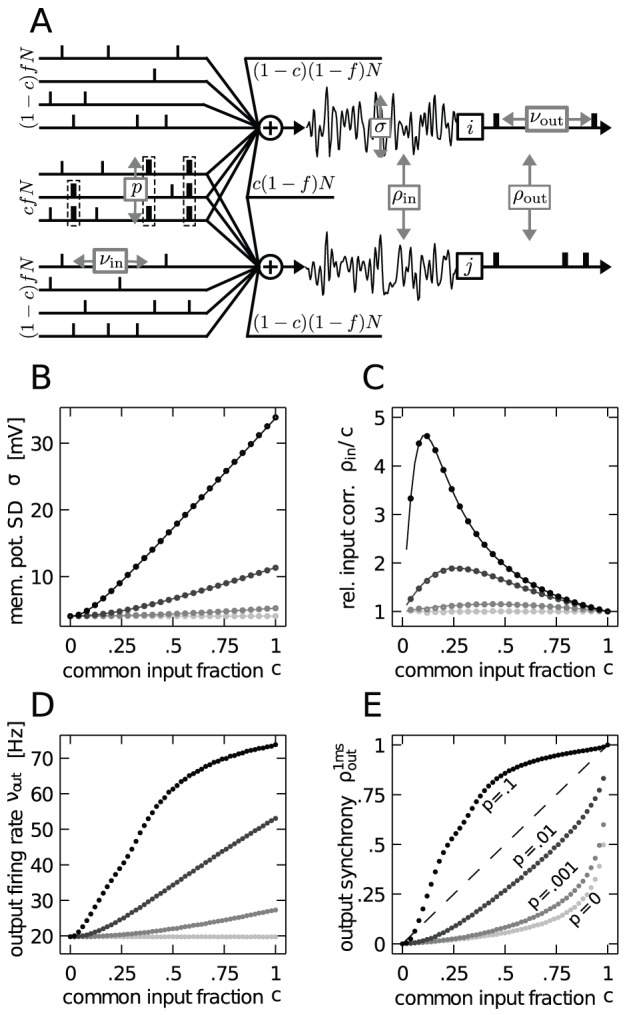
A pair of integrate-and-fire model neurons driven by partially shared and correlated presynaptic events. **A** Each of the neurons 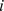 and 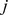 receives input from 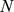 sources, of which 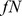 are excitatory and 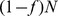 are inhibitory. Both neurons share a fraction 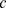 of their excitatory and inhibitory sources, whereas the fraction 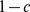 is independent for each neuron. Schematically represented spike trains on the left of the diagram show the excitatory part of the input; the inhibitory input is only indicated. A single source emits spike events with a firing rate 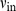, with marginal Poisson statistics. Correlated spiking is introduced in the 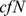 common excitatory sources to both neurons. This pairwise correlation is realized by means of a multiple interaction process (MIP) [39] that yields a correlation coefficient of 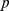 between any pairs of sources. In absence of a threshold, the summed input drives the membrane potential to a particular working point described by its mean 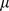 and standard deviation 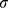 and the correlation coefficient 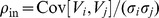 between the free membrane potentials 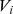, 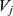 of both neurons. In presence of a threshold mean and variance of the membrane potential determine the output firing rate 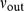 and their correlation in addition determines the output correlation 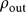, calculated by (2). **B**–**E** Direct simulation was performed using different values of common input fraction 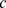 and four fixed values of input spike synchrony 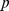 (as denoted in E). Each combination of 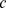 and 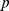 was simulated for 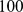 seconds; gray coded data points show the average over 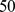 independent realizations. Remaining parameters are given in Table 1. Solid lines in B and C are calculated as (5) and (6), respectively. In C, for convenience, 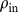 is normalized by the common input fraction 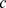, so that 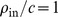 in absence of synchrony (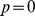). E shows the output spike synchrony 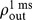.

The pivotal role of correlations distinguishing the two opposing views and the appearance of synchrony at task-specific times [17], [21], [22] suggests to ask the following question, illustrated in Fig. 1A: Can the experimentally observed synchrony between the activity of two neurons be explained solely by to the convergent connectivity with independently activated shared inputs or are in addition correlations among the afferents to both neurons required? If shared input is sufficient, synchrony is just a side effect of the convergent connectivity in the cortex. However, if synchronous activation of common afferents is required, it is likely that spike synchrony is used to propagate information through the network. A functional interpretation is assigned to synchrony by the picture of the cell assembly [13], [14], [31], [32], where jointly firing neurons dynamically form a functionally relevant subnetwork. Due to the local connectivity with high divergence and convergence, any pair of neurons shares a certain amount of input. This common input may furthermore exhibit spike synchrony, representing the coherent activity of the other members of the cell assembly. In the assembly picture, the synchronous input from peer neurons of the same assembly is thus considered conveying the signal, while theTe input from neurons outside of the assembly is considered as noise [33].

One particular measure for assessing the transmission of correlation by a pair of neurons is the transmission coefficient, i.e. the ratio of output to input correlation. When studying spiking neuron models, the synaptic input is typically modeled as Gaussian white noise, e.g. by applying the diffusion approximation to the leaky integrate-and-fire model [34]. In the diffusion limit, the transmission coefficient of a pair of model neurons receiving correlated input mainly depends on the firing rate of the neurons alone [35], [36]. For low correlations, linear perturbation theory well describes the transmission coefficient, which is always below unity, i.e. the output correlation is bounded by the input correlation, pairs of neurons always lose correlation [37]. Analytically tractable approximations of leaky integrate-and-fire neural dynamics have related the low correlation transmission to the limited memory of the membrane voltage [38]. The transmission is lowest if neurons are driven by excitation and inhibition, when fluctuations dominate the firing. In the mean driven regime the transmission coefficient can reach unity for integral measures of correlation [38].

Understanding the influence of synchrony among the inputs on the correlation transmission requires to extend the above mentioned methods, as Gaussian fluctuating input does not allow to represent individual synaptic events, not to mention synchrony. Therefore, in this work we introduce an input model that extends the commonly investigated Gaussian white noise model. We employ the multiple interaction process (MIP) [39] to generate an input ensemble of Poisson spike trains with a predefined pairwise correlation coefficient. We use these processes containing spike synchrony as the input common to both neurons and model the remaining afferents as independent Poisson spike trains. Furthermore, contrary to studies that measure the integrated output correlation (count correlation) [35], [36], we primarily consider the output correlation on the time scale of milliseconds, i.e. the type of correlation determined by the coincidence detection properties of neurons.

In section **“Results”** we first introduce the neuron and input models. In section **“Understanding and Isolating the Effect of Synchrony”** we study the impact of input synchrony on the firing properties of a pair of leaky integrate-and-fire neurons with current based synapses. Isolating and controlling this impact allows us to separately study the effect of input synchrony on the one hand and common input on the other hand on the correlation transmission. In section **“Correlation Transmission in the Low Correlation Limit”** and **“Correlation Transmission in the High Correlation Limit”** we present a quantitative explanation of the mechanisms involved in correlation transmission, in the limit of low and high correlation, respectively, and show how the transmission coefficient can exceed unity in the latter case. In section **“Discussion”** we summarize our findings in the light of previous research, provide a simplified model that enables an intuitive understanding and illustrates the generality of our findings. Finally, we discuss the limitations of our theory and consider possible further directions.

## Results

The neuronal dynamics considered in this work follows the leaky integrate-and-fire model, whose membrane potential 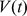 obeys the differential equation
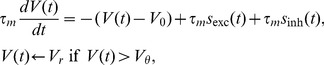
(1)where 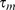 is the membrane time constant, 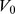 the resting potential, 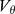 the firing threshold, and 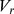 the reset potential of the neuron. The neuron is driven by excitatory and inhibitory afferent spike trains 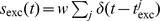 and 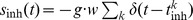 where 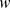 is the excitatory synaptic weight and 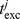 and 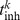 are the arrival time points of excitatory and inhibitory synaptic events, respectively. 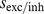 denote the weighted sum of all afferent excitatory and inhibitory events, respectively. Inhibitory events are further weighted by the factor 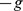. Each single incoming excitatory or inhibitory event causes a jump of the membrane potential by the synaptic weight 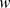 or 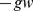, respectively, according to (1). Whenever the membrane potential reaches the threshold 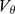 the neuron fires a spike and the membrane potential is reset to 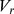 after which it is clamped to that voltage for a refractory period of duration 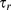. In the current work we measure the correlation between two spike trains 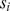 and 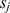 on the time scale 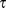 as
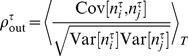
(2)where 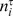 is the spike count of spike train 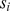 in a time window 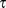 and the average 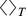 is performed over the 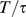 time bins of a stationary trial. In the current work we investigate correlations on two different time scales, 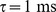 and 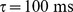, referred to in the following as 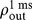 and 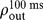, respectively.

We investigate the correlation transmission of a pair of neurons considering the following input scenario. Each neuron receives input from 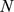 presynaptic neurons of which 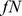 are excitatory and 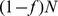 are inhibitory. Both neurons share a fraction 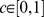 of their excitatory and inhibitory afferents. Fig. 1A shows a schematic representation of the input to neurons 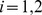. Each source individually obeys Poisson statistics with rate 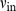. Our motivation to study this scenario comes from the idea of Hebbian cell assemblies [13], [14], [31], [32]. We imagine the considered pair of neurons to belong to an assembly. Both neurons receive 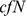 common excitatory inputs from peer neurons of the same group and 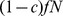 disjoint excitatory inputs from neurons possibly belonging to other assemblies. We further assume that synchronous firing of the assembly members is the signature of participation in an active assembly [13], [32]. We therefore ask how the synchronous activity among the 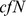 common excitatory inputs affects the correlation between the activity of the considered pair. In particular we choose a multiple interaction process (MIP) [39] to model the synchronous spike events in the common input. In this model each event of a mother Poisson process of rate 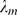 is copied independently to any of the 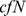 child spike trains with probability 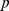, resulting in a pairwise correlation coefficient of 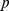 between two child spike trains. Thinking of the pair of neurons as a system that transmits a signal from its input to its output, we consider the Poisson events of the mother spike train as the signal, representing the points in time where a group of peer neurons of the assembly are activated. The disjoint inputs to both cells act as noise. By choosing the rate of the mother spike train as 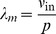 the rate of a single child spike train is 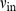 and independent of 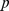.

Fig. 1B, C, D and E show that the amount of pairwise correlations in the common input has a strong impact on the variance and correlation of the free membrane potentials (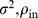) and therefore on the output firing rate and output spike synchrony (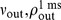). Let us first consider the case of 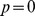, i.e. the absence of synchronous events in the input. As expected, the free membrane potential variance 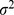 remains constant throughout the whole range of 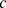, as does the firing rate 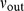 (Fig. 1B and D). Fig. 1C shows the correlation of the free membrane potential of a neuron pair, normalized by the common input fraction 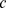. As expected, for 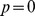 the input correlation is only determined by the common input fraction and thus 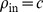. Hence, the output synchrony observed for 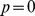 in Fig. 1E is solely due to the correlation caused by common input and describes the often reported correlation transmission function of the integrate-and-fire model [35], [36], where for 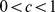 the output spike synchrony is always well below the identity line, which is in full agreement with the work of [35].

Let us now consider the case of 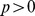. In Fig. 1B and D we observe that even small amounts of input synchrony result in an increased variance of the free membrane potential, which is accompanied by an increase of the output firing rate. While for weak input synchrony the increase of 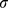 and 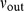 is only moderate, in the extreme case of strong input synchrony (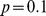) 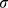 becomes almost ten-fold higher and 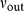 increases more than three-fold compared to the case of 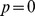. Fig. 1C shows that input synchrony also has a strong impact on the correlation between the free membrane potentials of a neuron pair. For any 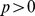 the input correlation is most pronounced for high 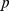 and in the lower regime of 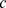. Simulation results shown in Fig. 1E suggest that this increase of input correlation is accompanied by an increased synchrony between the output spikes for 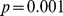 and 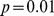. For strong input synchrony of 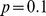 the output synchrony is always higher than the input correlation caused solely by the common input, except near 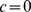 and at 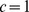.

The output firing rates and output spike synchrony shown in Fig. 1D and E bear a remarkable resemblance, most notably for lower values of 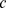. Particularly salient is the course of these quantities for 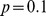, which is almost identical over the whole range of 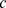. These observations clearly corroborate findings from previous studies that have shown an increase of the correlation transmission of a pair of neurons with the firing rate of the neurons [35], [36]. Thus, we must presume that a substantial amount of the output synchrony observed in Fig. 1E can be accounted for by the firing rate increase observed in Fig. 1D. Furthermore, as Fig. 1C suggests, for any 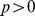 common input and the synchronous events both contribute to the correlation between the membrane potentials of a neuron pair.

### Understanding and Isolating the Effect of Synchrony

These two observations – the increase of input correlation and output firing rate induced by input synchrony – foil our objective to understand the sole impact of synchronous input events on the correlation transmission of neurons. In the following we will therefore first provide a quantitative description of the effect of finite sized presynaptic events on the membrane potential dynamics and subsequently describe a way to isolate and control this effect.

The synchronous arrival of 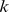 events has a 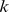-fold effect on the voltage due to the linear superposition of synaptic currents. The total synaptic input can hence be described by a sequence of time points 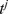 and independent and identically distributed (i.i.d) random number 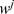 that assume a discrete set of synaptic amplitudes each with probability 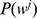. The train of afferent impulses follows Poisson statistics with some rate 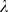. Assuming small weights 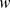 and high, stationary input rate 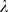, a Kramers-Moyal expansion [40], [41], [42] can be applied to (1) to obtain a Fokker-Planck equation for the membrane potential distribution 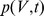


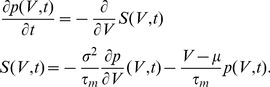
(3)Only the first two moments 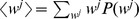 and 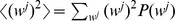 of the amplitude distribution enter the first (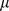) and second (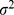) infinitesimal moments as [43], cf. Appendix Input–Output Correlation of an Integrate–and–Fire Neuron for a detailed derivation
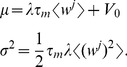
(4)In the absence of a threshold, the stationary density follows from the solution of 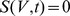 as a Gaussian with mean 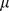 and variance 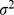.

Equation (3) and (4) hold in general for excitatory events with i.i.d. random amplitudes arriving at Poisson time points. Given the 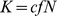 common excitatory afferents' activities are generated by a MIP process, the number of 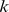 synchronized afferents follows a binomial distribution 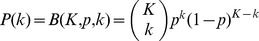, with moments 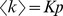 and 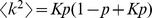. Note that throughout the manuscript we choose the number of common inputs 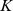 to be an integer, and we restrict the values of 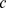 accordingly. The total rate 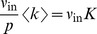 of arriving events is independent of 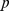, as is the contribution to the mean membrane potential 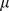. Further we assume the neurons to be contained in a network that is in the balanced state, i.e. 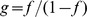, and that all afferents have the same rate 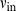. Thus, excitation and inhibition cancel in the mean so that 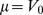. Due to the independence of excitatory and inhibitory spike trains they contribute additively to the variance 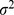 in (4). The variance due to 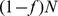 inhibitory afferents with rate 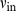 is 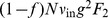, with 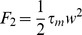. An analog expression holds for the contribution of unsynchronized disjoint excitatory afferents. The contribution of 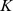 excitatory afferents from the MIP follows from (4) as 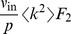. So together we obtain
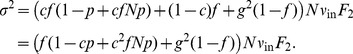
(5)


Fig. 1B shows that (5) is in good agreement with simulation results. We are further interested in describing the correlation 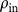 between the membrane potentials of both neurons. The covariance is caused by the contribution from shared excitation 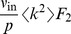, in addition to the contribution from shared inhibition 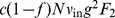, which together result in the correlation coefficient
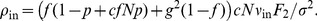
(6)


Again, Fig. 1C shows that (6) is in good agreement with simulation results.

In order to isolate and control the effect of the synchrony parameter 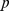 on the variance (5) and the input correlation (6), in the following we will compare two distinct scenarios: In the first scenario, common input alone causes the input correlation 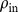 and spiking synchrony among afferents is zero (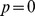). In the second scenario we generate the same amount of input correlation 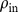 but realize it with a given amount of spike synchrony 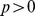. In order to have comparable scenarios, we keep the marginal statistics of individual neurons the same, measured by the membrane potential mean 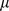 and variance 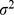.

In scenario 1 (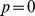) the input correlation 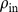 (6) equals the common input fraction 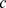. In scenario 2 (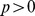) the same input correlation 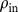 can be achieved by appropriately decreasing the fraction of common inputs to 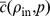. The value of 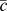 is determined by the positive root of the quadratic equation (6) solved for 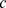. In neither scenario does the input correlation depend on the afferent rate 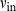. In scenario 2 we can hence choose 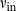 in order to arrive at the same variance 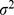 as in scenario 1. To this end we solve (5) for 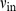 to obtain the reduced afferent rate 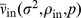.

**Figure 2:**
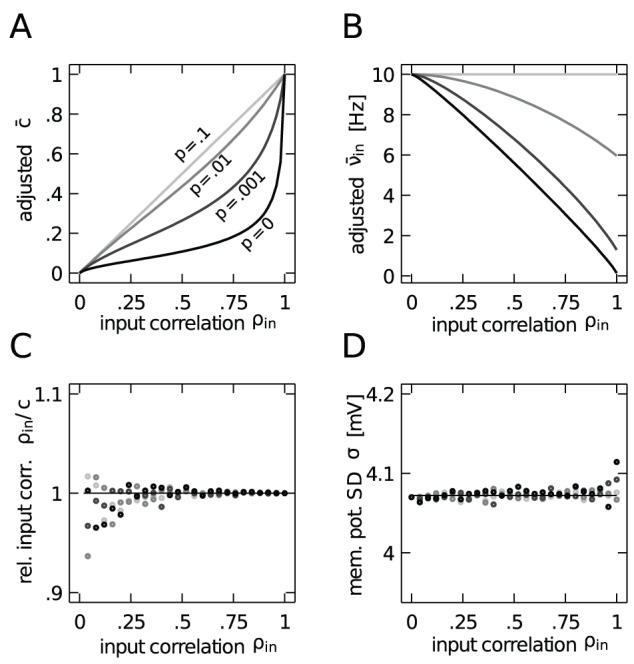
Isolation and control of the effect of synchrony on the free membrane potential statistics. **A**,**B** Adjusted common input fraction 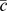 (**A**) and input firing rate 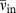 (**B**) for different values of 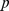 (gray coded) that ensure the same variance and covariance as for 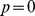. **C** Correlation coefficient 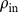 normalized by 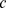 between the free membrane potential of a pair of neurons using the adjusted common input fraction 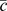. **D** Standard deviation of the free membrane potential, using the adjusted firing rate 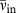. The statistics of the free membrane potential measured in simulations in panels C and D are further verified via (6) and (5) (solid lines).

We evaluate this approach by simulating the free membrane potential of a pair of leaky integrate-and-fire neurons driven by correlated input. For different values of 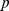 we chose 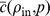 and 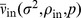, shown in Fig. 2A and B, to keep the variance and the correlation constant. Fig. 2A shows that the adjustment of the common input fraction becomes substantial only for higher values of 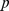: while for 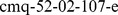 the reduced 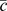 is only slightly smaller than 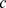, for 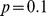 and 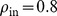 it is reduced to 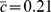. Fig. 2B shows that even for small amounts of input synchrony, 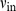 needs to be decreased considerably in order to prevent the increase of membrane potential variance (Fig. 1B). In the extreme case of 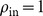 and 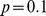 (both neurons receive identical and strongly synchronous excitatory input) an initial input firing rate of 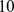 Hz needs to be decreased to 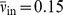 Hz. Fig. 2C and D confirm that indeed both the correlation and the variance of the free membrane potential remain constant throughout the whole range of 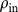 and for all simulated values of 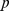.

### Correlation Transmission

**Figure 3:**
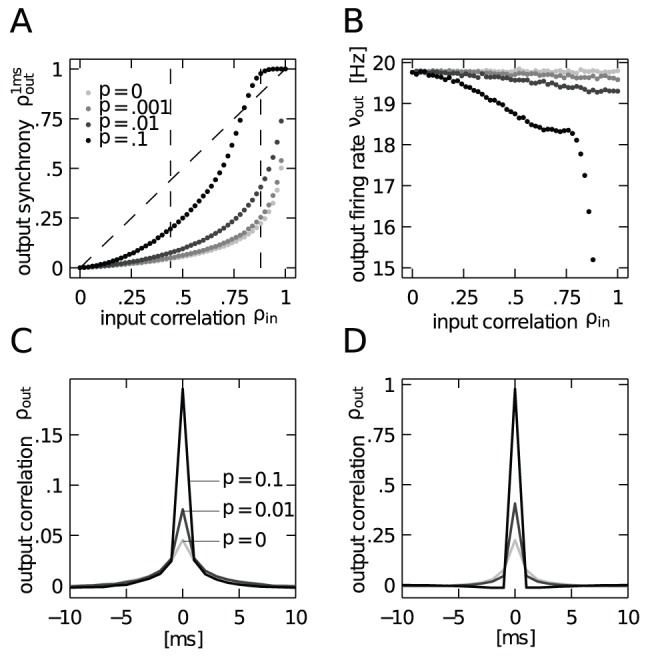
Correlation transmission of a pair of integrate-and-fire neurons. **A** Output spike synchrony as a function of input correlation 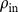 and for four different values of input synchrony 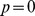, 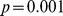, 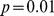 and 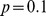 (gray-coded). Dashed black line with slope 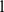 indicates 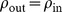. **B** Corresponding mean output firing rate of the neurons. **C**,**D** Cross-correlation functions at input correlations 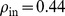 (C) and 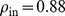 (D) (indicated by dashed vertical lines in A) for the three values of input synchrony 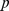 as indicated in C.

In order to study the transmission of correlation by a pair of neurons, we need to ensure that the single neuron's working point does not change with the correlation structure of the input. The diffusion approximation (3) suggests, that the decisive properties of the marginal input statistics are characterized by the first (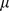) and second moment (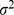). As we supply balanced spiking activity to each neuron, the mean 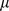 is solely controlled by the resting potential 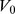, as outlined above. For any given value of 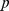 and 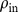, choosing the afferent rate 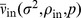 ensures a constant second moment 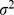. Consequently, Fig. 3 confirms that the fixed working point (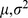) results in an approximately constant neural firing rate 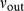 for weak to moderate input synchrony 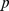. For strong synchrony, fluctuations of the membrane potential become non-Gaussian and the firing rate decreases; the diffusion approximation breaks down.

In studies which investigate the effect of common input on the correlation transmission of neurons, the input correlation is identical to the common input fraction 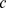 [35], [36]. In the presence of input synchrony this is obviously not the case (Fig. 1C). Choosing the afferent rate and the common input fraction according to 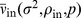 and 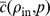, respectively, enables us to realize the same input correlation 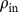 with different contributions from shared inputs and synchronized events. We may thus investigate how the transmission of correlation by a neuron pair depends on the relative contribution of synchrony to the input correlation 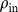. Fig. 3A shows the output synchrony as a function of 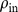 for four fixed values of input synchrony 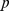. As the input correlation is by construction the same for all values of 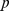, changes in the output synchrony directly correspond to a different correlation transmission coefficient. Even weak spiking synchrony (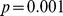) in the common input effectively increases the output synchrony, compared to the case where the same input correlation is exclusively caused by common input (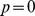). Stronger synchrony (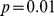 and 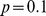) further increases this transmission. In Fig. 3B we confirm that the increase of output spike synchrony is not caused by an increase of the output firing rate of the neurons, but rather their rate remains constant up to intermediate values of 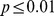. The drastic decrease of the output firing rate for 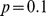 does not rebut our point, but rather strengthens it: correlation transmission is expected to decrease with lower firing rate [35], [36] for Gaussian inputs. However, here we observe the opposite effect in the case of strongly non-Gaussian inputs due to synchronous afferent spiking. We will discuss this issue in the following paragraph, deriving an analytical prediction for the correlation transmission. Moreover, we observe that the increased transmission is accompanied by a sharpening of the correlation function with respect to the case of 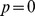 (cf. Fig. 3C and D).

For correlated inputs caused by common inputs alone (no synchrony, 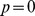) or by weak spiking synchrony (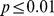) the transmission curves in Fig. 3A are always below the identity line. This means that the neural dynamics does not transmit the correlation perfectly, but rather causes a decorrelation. Recent work has shown that the finite life time of the memory stored in the membrane voltage of a leaky integrate-and-fire neuron is directly related to this decorrelation [38]. Quantitative approximations of this decorrelation by non-linear threshold units can be understood in the Gaussian white noise limit [37], [35], [36]. For input correlation caused by spiking synchrony, however, we observe a qualitatively new feature here. In the presence of strong spiking synchrony (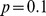), in the regime of high input correlation (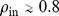) the correlation transmission coefficient exceeds unity. In other words, the neurons correlate their spiking activity at a level that is higher than the correlation between their inputs. In order to obtain a quantitative understanding of this boost of correlation transmission by synchrony, in the following two sections we will in turn investigate the mechanisms in the limit of low and high input correlations, respectively.

### Correlation Transmission in the Low Correlation Limit

In the limit of low input correlation Fig. 3 suggests that the main difference of the correlation functions is in the central peak caused by coincident firing of both cells. As the remainder of the covariance function only changes marginally, we can as well consider integral measures of the covariance function. Calculating the time integral of the covariance function can conveniently be accomplished by an established perturbative approach that treats the common input as a small perturbation and only requires the DC-susceptibility of the neuron to be determined [37], [44], [35], [36]. As the covariance function typically decays to zero on a time scale of about 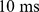, the integral correlation is well approximated by the covariance between spike counts in windows of 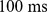, considered in this subsection.

For Gaussian white noise input and in the limit of low input correlation, the correlation transmission is well understood [37], [44], [35], [36]. The employed diffusion approximation assumes that the amplitudes of synaptic events are infinitesimally small. For uncorrelated Poisson processes and large number of afferents 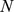, the theory is still a fairly good approximation. For small synaptic jumps approximate extensions are known [45], [46] and exact results can be obtained for jumps with exponentially distributed amplitudes [47]. However, in order to treat spiking synchrony in the common input to a pair of neurons, we need to extend the perturbative approach here.

**Figure 4:**
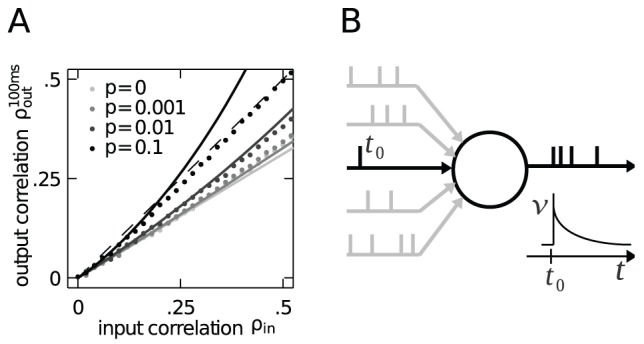
Approximation of the output correlation in the limit of low input correlation. **A** Correlation transmission in the low input correlation limit. Data points show the output correlation 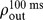 resulting from simulations, solid lines show the theoretical approximation 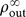 (10). Dashed black line indicates 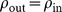. **B** Deflection of the firing rate with respect to base rate caused by an additional synaptic event at 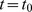.

Before deriving an expression for the correlation transmission by a pair of neurons, we first need the firing rate deflection of a neuron 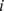 caused by a single additional synaptic impulse of amplitude 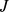 at 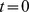 on top of synaptic background noise. Within the diffusion approximation, the background afferent input to the neuron can be described by the first two moments 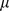 and 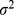 (4). We denote as 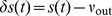 the centralized (zero mean) spike train and as 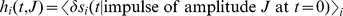 the excursion of the firing rate of neuron 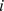 with respect to the base rate 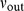 caused by the additional impulse and averaged over the realizations of the background input 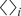, illustrated in Fig. 4B. An additional impulse is equivalent to an instantaneous perturbation of both, the first (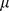) and the second (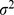) moment with prefactors 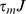 and 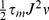, respectively, as shown in section **“Impulse Response to Second Order”**. The DC-susceptibility 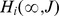 is therefore a quadratic function in the amplitude 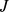


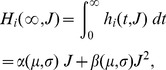
(7)where the prefactors 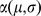 and 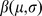 depend on the working point of the neuron and hence on the background noise parameterized by 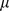 and 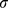. A similar approximation to second order in 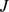 was performed for periodic perturbations of the afferent firing rate [48, cf. Appendix, eq. A3] and for impulses in [12, cf. App. 4.3 and Fig. 8 for an estimate of the validity of the approximation]. Note that this approximation extends previous results that are first order in 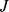 [49], [46]. The DC-susceptibility 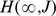 can be interpreted as the expected number of additional spikes over baseline caused by the injected pulse of amplitude 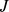. As the marginal statistics of the inputs to both neurons are the same they fire with identical rates. Each commonly received impulse to both cells contributes to the cross covariance function between the outgoing spike trains, defined as
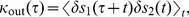
(8)where the expectation value 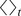 is taken over realizations of the disjoint inputs, the common input, and over time 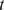. 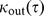 drops to zero for 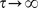. The average over realizations of the afferent input ensembles can be performed separately over realizations of the common 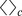 and the disjoint inputs 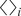, 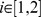 [49], leading to
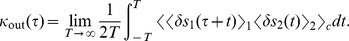
Transforming to frequency domain with respect to 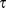 and applying the Wiener-Khinchine theorem [50], the cross spectrum between the centralized spike trains reads
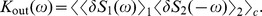
With the definition of the Fourier transform 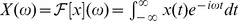, for 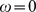 the cross spectrum equals the time integral of the cross correlation function. Performing the average 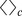 over the common sources we obtain two contributions, due to synchronous excitatory pulses from the MIP [39], giving rise to 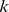 synchronously arriving events, 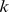 being distributed according to a binomial distribution 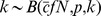, and due to 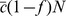 common inhibitory inputs each active with Poisson statistics and rate 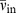, leading to
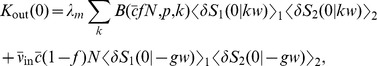
where 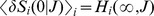 is the integral of the response to a single impulse of amplitude 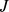. So with (7) we have 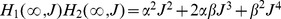 and finally obtain
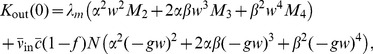
(9)where 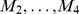 are the moments of the binomial distribution (Section **“Moments of the Binomial Distribution”**). In order to obtain a correlation coefficient, we need to normalize the integral of (9) by the integral of the auto-covariance of the neurons' spike trains. This integral equals 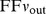 [51], [44], with the Fano factor 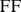. In the long time limit the Fano factor of a renewal process equals the squared coefficient of variation 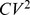 [52], which can be calculated in the diffusion limit [40, App. A1]. Thus, we obtain
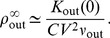
(10)
Fig. 4A shows that the output spike correlation of a pair of neurons is fairly well approximated by 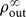 in the lower correlation regime. While the approximation is good over almost the whole displayed range of 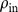 for 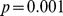 and 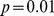, for 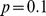 the theory only works for values of 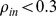 in agreement with previous studies [35], [36] applying a similar perturbative approach to the case of Gaussian input fluctuations.

### Correlation Transmission in the High Correlation Limit

**Figure 5:**
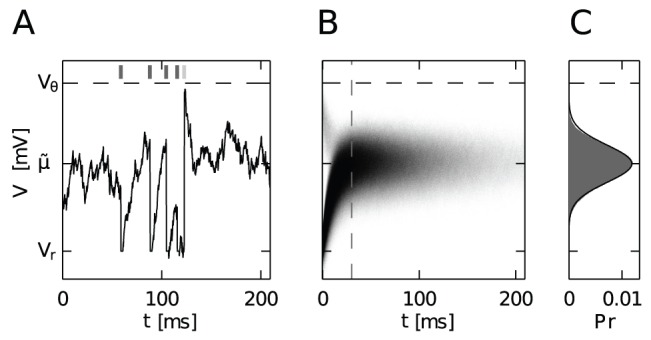
Neural dynamics in the regime of high input correlation and strong synchrony. **A** Exemplary time course of a membrane potential driven by input containing strong, synchronous spike events. During the time period shown, five MIP events arrive (indicated by tick marks above 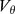). The first four drive the membrane potential above the threshold 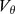, after which 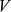 is reset to 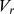 and the neuron emits a spike (dark gray tick marks above 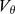). The fifth event is not able to deflect 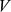 above threshold (light gray) and the membrane potential quickly repolarizes towards its steady state mean 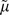 (see text). **B** Time-resolved membrane potential probability density 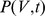 triggered on the occurrence of a MIP event at 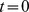. Since most MIP events elicit a spike, after resetting 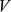 to 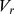 the membrane potential quickly depolarizes and settles to a steady state Gaussian distribution. The slight shade of gray observable for small 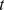 just below the threshold 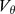 is caused by the small amount of MIP events that were not able to drive the membrane potential above threshold. **C** Probability density of the membrane potential in steady state. Theoretical approximation (black) was computed using 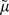 and 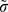 (see text and eq:Vt), empirical measurement (gray) was performed for 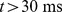 (gray dashed line in B). Simulation parameters were 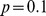, 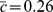 (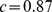) and 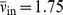 (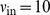) Hz. Other parameters as in Table 1.

In order to understand how the neurons are able to achieve a correlation coefficient larger than one, we need to take a closer look at the neural dynamics in the high correlation regime. We refer to the strong pulses caused by synchronous firing of numerous afferents as MIP events. Fig. 5A shows an example of the membrane potential time course that is driven by input in the high correlation regime. At sufficiently high synchrony as shown here, most MIP events elicit a spike in the neuron, whereas fluctuations due to the disjoint input alone are not able to drive the membrane potential above threshold. Thus, in between two MIP events the membrane potential distribution of each neuron evolves independently and fluctuations are caused by the disjoint input alone. Fig. 5B shows the time-dependent probability density of the membrane potential, triggered on the time of arrival of a MIP event. We observe that most MIP events cause an action potential, followed by the recharging of the membrane after it has been reset to 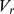 at 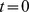. After a short period of repolarization the membrane potential quickly reaches its steady state. The contribution of the 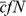 common, excitatory afferents to the membrane potential statistics is limited to those occasional strong depolarizations. Between two such events they neither contribute to the mean nor to the variance of 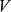. Hence the effective mean and variance of the membrane potential are due to the disjoint input alone, given by 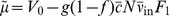 and 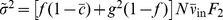 with 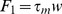 and 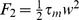. Fig. 5C shows in gray the empirical distribution of the membrane potential between two MIP events after it has reached the steady state. It is well approximated by a Gaussian distribution with mean 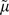 and variance 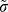. The membrane potential can therefore be approximated as a threshold-free Ornstein-Uhlenbeck process [53], [54].

Let us now recapitulate these last thoughts in terms of a pair of neurons: In the regime of synchronized high input correlation (e.g. 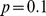, 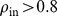), MIP events become strong enough so that most of them elicit a spike in both neurons. At the same time, the uncorrelated, disjoint sources (which can be considered as sources of noise) induce fluctuations of the membrane potential which are, however, not big enough to drive the membrane potential above threshold. Thus, while the input to both neurons still contains a considerable amount of independent noise, their output spike trains are (for sufficiently high 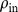) a perfect duplicate of the mother spike train that generates the MIP events in their common excitatory input, explaining the observed correlation transmission coefficient larger than unity. Note that this is the reason for the drastic decrease of the output firing rate in Fig. 3B, which in the limit of high input correlation approaches the adjusted input firing rate 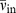 (Fig. 2B).

We would like to obtain a qualitative assessment of the correlation transmission in the high correlation input regime. Since the probability of output spikes caused by the disjoint sources is vanishing, the firing due to MIP events inherits the Poisson statistics of the mother process. Consequently, the auto-covariance function of each neurons' output spike train is a 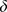-function weighted by its rate 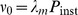, where 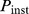 is the probability that a MIP event triggers an outgoing spike in one of the neurons. The output correlation can hence be approximated by the ratio
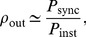
(11)where 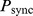 is the probability that a MIP event triggers an outgoing spike in both neurons at the same time. Note that the approximation (11) holds for arbitrary time scales, as the spike trains have Poisson statistics in this regime. In order to evaluate 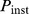 and 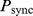, we use the simplifying assumption that the last MIP event at 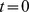 caused a reset of the neuron to 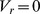, so the distribution 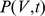 of the membrane potential evolves like an Ornstein-Uhlenbeck process as [54]

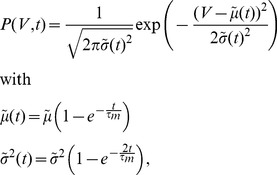
(12)which is the solution of (3) with initial condition 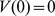. We evaluate 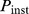 from the probability mass of the voltage density shifted across threshold by an incoming MIP event as
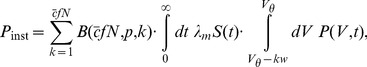
(13)where the survivor function 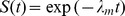 is the probability that after a MIP event occurred at 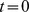 the next one has not yet occurred at 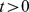. So 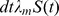 is the probability that no MIP event has occurred in 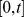 and it will occur in 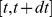 [52]. The binomial factor 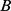 is the probability for the amplitude of a MIP event to be 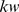 and the last integral is the probability that a MIP event of amplitude 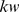 causes an output spike [46]. We first express 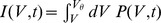 in terms of the error function using (12) with the substitution 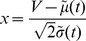, to obtain
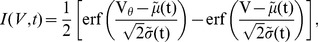
(14)where we used the definition of the error function 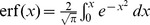. We further simplify the first integral in (13) with the substitution 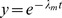 to
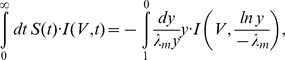
thus finally obtaining
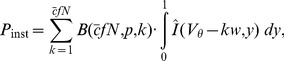
(15)where we introduced 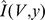 as a shorthand for (14) with 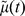 and 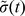 expressed in terms of the substitution variable 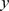 as 
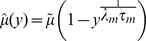
 and 
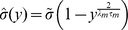
, following from (12). In order to approximate the probability 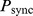 that the MIP event triggers a spike in both neurons we need to square the second integral in (13), because the voltages driven by disjoint input alone are independent, so their joint probability distribution factorizes, leading to
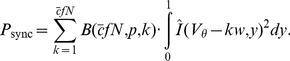
(16)It is instructive to observe that 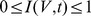, because 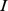 given by (14) is a probability. Therefore it follows that 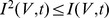, with equality reached if 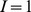 or 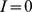. Hence from the definitions (15) and (16) it is obvious that 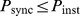, as it should be and the ratio (11) defines a properly bounded correlation coefficient 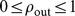 in the high input correlation regime.

**Figure 6:**
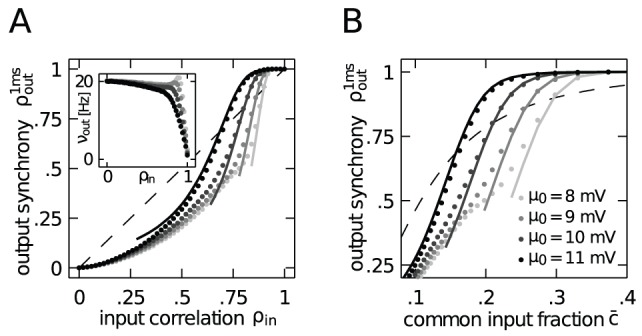
Approximation of the output synchrony in the limit of high input correlation. **A** Output spike synchrony as a function of input correlation in the limit of high input correlation and strong synchrony 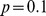. Data points and solid lines show results from simulations and theoretical approximation (11), respectively. Gray code corresponds to the four different mean membrane potential values 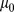 as depicted in B, the input firing rate 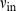 was 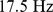, 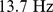, 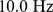 and 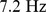, correspondingly (from low to high 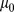). The working point used in the previous sections corresponds to 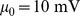, 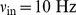. The inset shows the output firing rate at the four working points. **B** Output spike synchrony as a function of the actual common input fraction 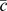 at the four working points. Dashed curves in A and B indicate 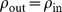.

So far, we have considered both neurons operating at a fixed working point, defined by the mean and variance (4). Due to the non-linearity of the neurons we expect the effect of synchronous input events on their firing to depend on the choice of this working point. We therefore performed simulations and computed (2) using four different values for the mean membrane potential 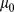 (Fig. 6). This was achieved by an appropriate choice of a DC input current 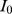 and accordingly adjusting the input firing rate 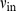 in order to keep the mean firing rate constant (Fig. 6A, inset). The data points from simulations in Fig. 6A show that different working points of the neurons considerably alter the correlation transmission in the limit of high input correlation. At working points near the threshold (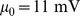) MIP events more easily lead to output spikes, thereby boosting the transmission of correlation, as compared to working points that are further away from the threshold (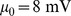). Solid lines in Fig. 6A furthermore show that (11) indeed provides a good approximation of the output spike correlation when the input to both neurons is strongly synchronized. Obviously, the assumption has to hold that the probability density of the membrane potential is sufficiently far from the threshold, which for 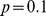 is only the case if 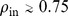. Hence, the approximation becomes less accurate for lower input correlations, as expected. Note that, as opposed to Fig. 1E, the effective common input fraction 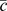 in Fig. 6A is much lower than 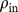. Fig. 6B shows the same data as a function of the actual fraction of shared afferents 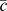. It reveals that the gain of correlation transmission above unity is already reached at fractions of common input as low as 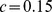 (for 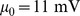), which is a physiologically plausible value.

A further approximation of (15) and (16) confirms the intuitive expectation that the mean size of a synchronous event compared to the distance of the membrane potential to the threshold plays an important role for the output synchrony: if synchrony is sufficiently high, say 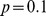, the binomial distribution 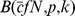 is rather narrow and has a peak at 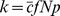. Inserting this mean value into (15) and (16) we obtain the approximation
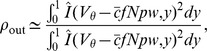
which shows that the response probability at time 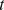 after a spike mainly depends on 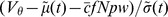.

**Figure 7:**
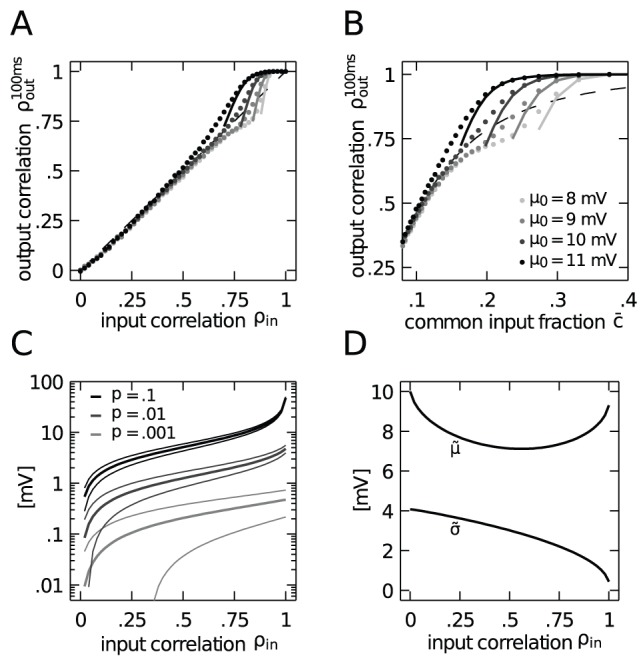
Correlation transmission for the output correlation on a long time scale 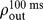 in the presence of strong input synchrony (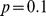). **A**,**B** As Fig. 6A,B but measuring the spike count correlation between the neurons over a time window of 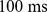. **C** Mean 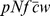 (thick lines) and mean plus minus one standard deviation 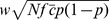 (thin lines) of the amplitude of synchronous spike volleys in the common excitatory input as a function of 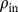 for three different values of 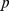 (indicated by gray code). **D** Mean 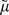 and standard deviation 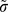 of the membrane potential caused solely by the disjoint afferents for strong synchrony (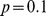) as a function of 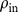.

Measuring the integral of the output correlation over a window of 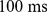, in the limit of high input correlation 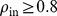 and strong synchrony 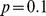 the picture qualitatively stays the same. Spikes are predominantly produced by the strong depolarizations caused by the synchronously arriving impulses. The output spike trains hence inherit the Poisson statistics from the arrival times of the synchronous volleys. As for marginal Poisson statistics and exactly synchronous output spikes the correlation coefficient does not depend on the time window over which the correlation is measured, the output correlation coefficient is uniquely determined by the ratio of the rates that both neurons fire together over the rate of each neuron firing individually, expressed by (11). This theoretical expectation is shown in Fig. 7A and B to agree well with the simulation results for different values of the mean membrane potential.

A qualitatively new behavior is observed in the intermediate range of input correlation 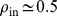: the input correlation is transmitted faithfully to the output with a gain factor around unity. Note that in the absence of synchrony the correlation gain is strictly below unity, as shown in Fig. 4. In the following we consider the point 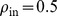 to provide a qualitative argument explaining the unit gain. Fig. 7C shows the average postsynaptic amplitude caused by a volley of synchronously arriving impulses 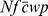, which is about 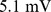 fluctuating only weakly with a small standard deviation of 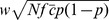 around 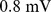. Fig. 7D shows that the mean membrane potential due to the disjoint input alone is around 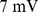, so two synchronous impulses closely appearing in time are sufficient to fire the neuron. Moreover, the fluctuations 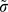 caused by the disjoint afferents alone are strong (around 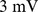) and with the mean membrane potential 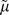 of around 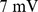 they are sufficient to fire the cell. As the integral over the covariance function equals the count covariance over long windows of observation 
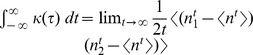
, we consider the spike counts 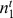 and 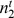 in a long time window 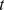. As each source of fluctuations (disjoint and common inputs) alone is already sufficient to fire the cell, both sources mutually linearize the neuron. Averaging the deviation of the spike count from baseline 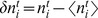 separately over each source of noise (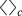 over common, 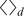 over disjoint sources) this deviation can be related linearly to the fluctuation of the respective other source, 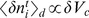, 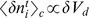. If such a linear relationship holds, it is directly evident that correlations are transmitted faithfully
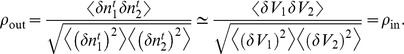



**Figure 8:**
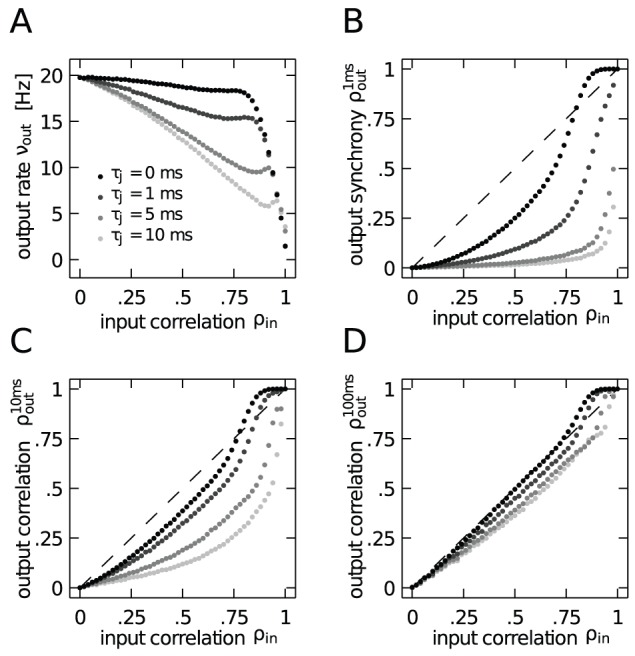
Correlation transmission for strong synchrony (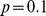) with jittered spike volleys. Panels show simulation results using 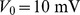 and four different jitter widths 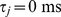, 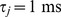, 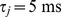 and 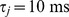 (gray code as shown in panel A). **A** Output firing rate as a function of input correlation for different jitter widths. **B**–**D** Output correlations 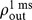 (B), 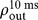 (C) and 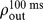 (D) as a function of the input correlation for increasing jitter widths.

So far, for 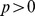 we have considered the case of input events in the common excitatory input that are perfectly synchronized. In the following we investigate how the transmission of strong synchrony 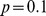 changes if the common excitatory input events are not perfectly synchronous by randomly jittering the spike times in each volley according to a normal distribution with a standard deviation 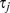. Fig. 8A shows that increasing the temporal jitter of the spike volleys results in a decrease of the mean output firing rate of neurons, in line with the decrease of the input variance caused by the jittering. Fig. 8B shows that also the output synchrony 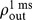 between the neurons is substantially decreased with increasing jitter 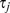. This observation is the result of three consequences of the jitter. Firstly, from the decreased firing rate observed in Fig. 8A we expect the correlation transmission to decrease [35], [36]. Secondly, due to the measurement of output synchrony on the precise time scale of 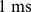, every dispersion of the input spikes exceeding this time window lowers the output correlation. Thirdly, for a jitter width comparable to the membrane time constant the leak term of the integrate-and-fire neuron reduces the summed effect of the input spikes on the membrane potential the more, the stronger the dispersion of the spike times. Thus, when considering the output synchrony 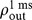 even with a jitter as small as 1 ms the case of 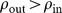 is not reached in the regime of high input correlation. However, on longer correlation time windows (Fig. 8C, D) a correlation gain 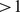 is possible with jitter widths up to 5 ms. This is intuitively expected, because spikes arriving within a short time interval compared to the membrane time constant (here 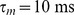) have in sum the same effect as if arriving in synchrony. Thus, measuring the output correlation on the same time scale as the jitter ‘collects’ this cumulative effect.

## Discussion

### Summary of Results

In this work we investigate the correlation transmission by a neuron pair, using two different types of input spike correlations. One is caused solely by shared input – typically modeled as Gaussian white noise in previous studies [35], [36] – while in the other the spikes in the shared input may additionally arrive in synchrony. In order to shed light on the question whether cortical neurons operate as integrators or as coincidence detectors [18], [24], [25], we investigate their efficiency in detecting and transmitting spike correlations of either type. We showed that the presence of spike synchrony results in a substantial increase of correlation transmission, suggesting that synchrony is a prerequisite in explaining the experimentally observed excess spike synchrony [17], [21], [22], rather than being an epiphenomenon of firing rate due to common input given by convergent connectivity [8].

To model correlated spiking activity among the excitatory afferents in the input to a pair of neurons we employ the Multiple Interaction Process (MIP) [39], resulting in non-Gaussian fluctuations in the membrane potential of the receiving neurons. In this model the parameter 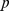 defines the pairwise correlation coefficient between each pair of 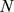 spike trains. If 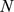 is large enough and all spike trains are drawn independently (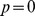) the summation of all 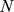 spike trains is approximately equivalent to a Gaussian white noise process [41], [54]. However, introducing spike correlations between the spike trains (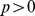) additionally allows for the modeling of non-Gaussian fluctuating inputs. Such correlations have a strong effect on the membrane potential statistics and the firing characteristics of the neurons. The fraction of common input 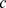 and the synchrony strength 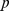 each contribute to the total correlation between the inputs to both neurons. We show how to isolate and control the effect of input synchrony such that (1) a particular input correlation 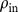 can be realized by an (almost) arbitrary combination of input synchrony 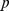 and common input fraction 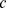, and (2) the output firing rate of the neurons does not increase with 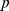. This enables a fair comparison of transmission of correlation due to input synchrony and due to common input. We find that the non-linearity of the neuron model boosts the correlation transmission due to the strong fluctuations caused by the common source of synchronous events.

Given a fixed input correlation, the correlation transmission increases with 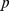. Most notably, this is the case although the output firing rate of the neurons does not increase and is for the most part constant, suggesting that the correlation susceptibility of neurons is not a function of rate alone, as previously suggested [35], but clearly depends on pairwise synchrony in the input ensemble. Previous studies have shown how to apply Fokker-Planck theory and linear perturbation theory to determine this transmission of correlation by pairs of neurons driven by correlated Gaussian white noise [37], [44], [35], [36]. In order to understand the effect of synchrony on the correlation transmission here we extended the Fokker-Planck approach to synaptic input of finite amplitudes. In the limit of low input correlation this extension indeed provides a good approximation of the output correlation caused by inputs containing spike correlations. Alternative models that provide analytical results are those of thresholded Gaussian models [55], [56] or random walk models [38]. In order to study transmission in networks with different architecture than the simple feed-forward models employed here, our results may be extended by techniques to study simple network motifs developed in [57].

Hitherto existing studies argue that neurons either loose correlation when they are in the fluctuation driven regime or at most are able to preserve the input correlation in the mean driven regime [58]. Here, we provide evidence for a qualitatively new mechanism which allows neurons to exhibit more output correlation than they receive in their input. Fig. 3A and Fig. 7A show that in the regime of high input correlation the correlation transmission coefficient can exceed unity. This effect, observed at realistic values of pairwise correlations (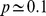) and common input fractions (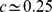), does not depend on the time scale of the measure of output spike correlation and furthermore withstands a jittering of the input synchrony up to the time scale of the membrane time constant. This time scale is on the same order as the experimentally observed dynamically changing precision of synchrony [59], accessible through theoretical and methodological advances to determine and detect significant spike synchrony [19], [60]. We provide a quantitative explanation of the mechanism that enables neurons to exhibit this behavior. We show that in this regime of high input correlation 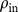 the disjoint sources and the common inhibitory sources do not contribute to the firing of the neurons, but rather the neurons only fire due to the strong synchronous events in the common excitatory afferents. Based on this observation, we derive an analytic approximation of the resulting output correlation beyond linear perturbation theory that is in good agreement with simulation results.

### Mechanism of Noise Suppression by Coincidence Detection

**Figure 9:**
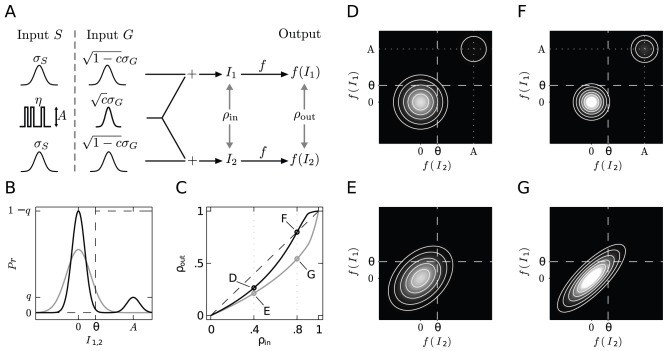
Mechanistic model of enhanced correlation transmission by synchronous input events. **A** The detailed model discussed in the results section is simplified two-fold. 1) We consider binary neurons with a static non-linearity 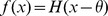. 2) We distinguish two representative scenarios with different models for the common input: 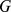: Gaussian white noise with variance 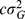, representing the case without synchrony, or 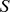: a binary stochastic process 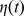 with constant amplitude 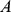, mimicking the synchronous arrival of synaptic events. In both scenarios in addition each neuron receives independent Gaussian input. **B** Marginal distribution of the total input 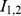 to a single neuron for input 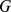 (gray) and 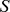 (black) and for 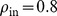. In input 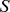 the binary process 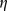 alternates between 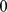 (with probability 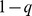) and 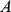 (with probability 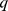), resulting in a bimodal marginal distribution. The mean activity of one single neuron is given by the probability mass above threshold 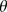. We choose the variances 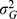 and 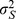 of the disjoint Gaussian fluctuating input such that the mean activity is the same in both scenarios. **C** Output correlation 
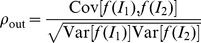
 as a function of the input correlation 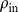 (see A) between the total inputs 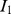 and 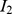. Probability 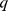 is chosen such that inputs 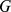 and 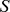 result in the same input correlation 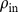. The four points marked by circles correspond to the panels D–G. **D**–**G** Joint probability density of the inputs 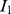, 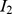 to both neurons. For two different values of 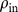 the lower row (E,G) shows the scenario 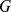, the upper row (D,F) the scenario 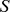. Note that panel B is the projection of the joint densities in F and G to one axis. Brighter gray levels indicate higher probability density; same gray scale for all four panels.

We presented a quantitative description of the increased correlation transmission by synchronous input events for the leaky integrate-and-fire model. Our analytical results explain earlier observations from a simulation study modeling synchrony by co-activation of a fixed fraction of the excitatory afferents [61]. However, the question remains what the essential features are that cause this effect. An even simpler model consisting of a pair of binary neurons is sufficient to qualitatively reproduce our findings and to demonstrate the generality of the phenomenon for non-linear units, allowing us to obtain a mechanistic understanding. In this model, whenever the summed input 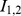 exceeds the threshold 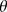 the corresponding neuron is active (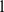) otherwise it is inactive (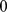). In Fig. 9 we consider two different implementations of input correlation, one using solely Gaussian fluctuating common input (input 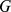), the other representing afferent synchrony by a binary input common to both neurons (input 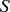). The binary stochastic signal 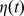 has value 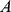 with probability 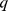 and 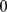 otherwise, drawn independently for successive time bins. Background activity is modeled by independent Gaussian white noise in both scenarios. The input 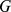 corresponds to the simplified model presented in [35, cf. Fig.4] that explains the dependence of the correlation transmission of the firing rate. In order to exclude this dependence, throughout Fig. 9 we choose the parameters such that the mean activity of the neurons remains unchanged. As shown in the marginal distribution of the input current to a single neuron in Fig. 9B, in the scenario 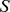 the binary process 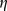 causes an additional peak with weight 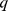 centered around 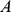. Equal activity in both scenarios requires a constant probability mass above threshold 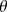, which can be achieved by an appropriate choice of 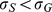. In scenario 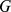 the input correlation equals the fraction of shared input 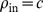, as in [35], whereas in scenario 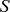 the input correlation is 
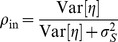
, where 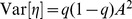 is the variance of the binary input signal 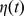. Comparing both scenarios, in Fig. 9C–G we choose 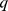 such that the same input correlation is realized.

As for our spiking model, Fig. 9C shows an increased correlation transmission due to input synchrony. This observation can be intuitively understood from the joint probability distribution of the inputs (Fig. 9D–G). Whenever any of the inputs exceeds the threshold (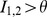) the corresponding neuron becomes active, whenever both inputs exceed threshold at the same time (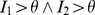), both neurons are synchronously active. Therefore, 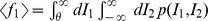, the probability mass on the right side of 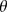 for input 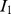 (corresponding definition for 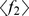), is a measure for the activity of the neurons. Analogously, 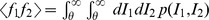, the probability mass in the upper right quadrant above both thresholds is a measure for the output correlation between both neurons. Since by our model definition the mean activity of both neurons is kept constant, the masses 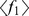 and 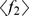 are equal in all four cases. However, the decisive difference between scenarios with inputs 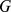 and 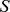 is the proportion of 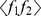 on the total mass above threshold 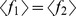. This proportion is increased by the common synchronous events, observable by comparing Fig. 9D,E. The more this proportion approaches 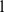, 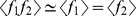, the more the activity of both neurons is driven by 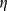 (Fig. 9F). At the same time the contribution of the disjoint fluctuations on the output activity is more and more suppressed. As the correlation coefficient relates the common to the total fluctuations, the correlation between the outputs can exceed the input correlation if the transmission of the common input becomes more reliable than the transmission of the disjoint input (cf. point marked as F in Fig. 9C).

The situation illustrated in Fig. 9 is a caricature of signal transmission by a pair of neurons of a cell assembly. The signal of interest among the members of the assembly consists of synchronously arriving synaptic events from peer neurons of the same assembly. In our toy model such a volley is represented by an impulse of large amplitude 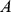. The remaining inputs are functionally considered as noise and cause the dispersion of 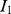 and 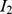 observable in Fig. 9D–F. In the regime of sufficiently high synchrony (corresponding to large 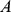) in Fig. 9F, the noise alone rarely causes the neurons to be activated, it is suppressed in the output signal due to the threshold. The synchrony coded signal, however, reliably activates both neurons, moving 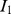 and 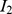 into the upper right quadrant. Thus a synchronous volley is always mapped to 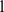 in the output, irrespective of the fluctuations caused by the noise. In short, the non-linearity of neurons suppresses the noise in the input while reliably detecting and transmitting the signal. A similar effect of noise cancellation has recently been described to prolong the memory life-time in chain-like feed forward structures [62].

### Limitations and Possible Extensions

Several aspects of this study need to be taken into account when relating the results to other studies and to biological systems. The multiple interaction process as a model for correlated neural activity might seem unrealistic at first sight. However, a similar correlation structure can easily be obtained from the activity of a population of 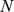 neurons. Imagine each of the neurons to receive a set of uncorrelated afferents causing a certain mean membrane potential 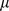 and variance 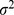. The entire population is then described by a membrane potential distribution 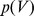. In addition, each neuron receives a synaptic input of amplitude 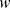 that is common to all neurons. Whenever this input carries a synaptic impulse, each of the 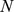 neurons in the population has a certain probability to emit a spike in direct response. The probability equals the amount of density shifted across threshold by the common synaptic event. Given the value 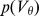 and its slope 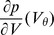 of the membrane potential density at threshold 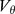, the response probability is 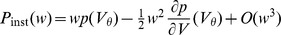 to second order in the synaptic weight 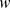. Employing the diffusion approximation to the leaky integrate-and-fire neuron, the density vanishes at threshold 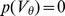 and the slope is given by 
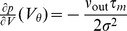
 [34]. The response probability hence is 
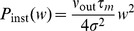
. For typical values of 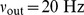, 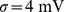, and 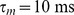 the estimate yields 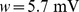 to get the copy probability 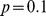 used in the current study. Such a synaptic amplitude is well in the reported range for cortical connections [28]. As each of the neurons within the population responds independently, the resulting distribution of the elicited response spikes is binomial, as assumed by the MIP. Moreover, since our theory builds on top of the moments of the complexity distribution it can be extended to other processes introducing higher order spike correlations [61], [39].

The correlation transmission coefficient can only exceed unity if the firing of the neurons is predominantly driven by the synchronously arriving volleys and disjoint input does not contribute to firing. The threshold then acts as a noise gate, small fluctuations caused by disjoint input do not penetrate to the output side. In the mean driven regime, i.e. when 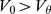, this situation is not given since every fluctuation in the input either advances (excitatory input) or delays (inhibitory input) the next point of firing. Especially at high firing rates the ‘forgetting’ of the fluctuation due to the leak until the next firing can be neglected, the leaky integrate-and-fire neuron behaves like a perfect integrator. Perfect integrators transmit fluctuations linearly, so 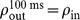 [58]. Given strong input synchrony (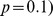 and 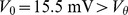, simulation results show that in the regime up to input correlations 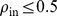 the neurons exhibit such a linear transmission (data not shown). For 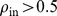 the correlation transmission decreases as the firing rate substantially decreases in the regime of high 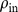. This smaller firing rate moves the dynamics away from the perfect integrator as the neurons loose more memory about the commonly received pulses between two spikes.

The boost of output correlation by synchronous synaptic impulses relies on fast positive transients of the membrane potential and strong departures from the stationary state: An incoming packet of synaptic impulses brings the membrane potential over the threshold within short time. Qualitatively, we therefore expect similar results for short, but non-zero rise times of the synaptic currents. For long synaptic time constants compared to the neuronal dynamics, however, the instantaneous firing intensity follows the modulation of the synaptic current adiabatically [44], [63]. A similar increase of output synchrony in this case can only be achieved if the static 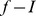 curve of the neuron has a significant convex non-linearity.

The choice of the correlation measure is of importance when analyzing spike correlations. It has been pointed out recently that the time scale 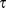 on which spike correlations are measured is among the factors that can systematically bias correlation estimates [3]. In particular, spike count correlations computed for time bins larger than the intrinsic time scale of spike synchrony can be an ambiguous estimate of input cross correlations [64]. Considering the exactly synchronous arrival of input events generated by the MIP, we chose to measure count correlations on a small time scale of 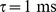 as well as on a larger scale of 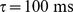.

### Conclusion

It has been proposed that the coordinated firing of cell assemblies provides a means for the binding of coherent stimulus features [14], [15], [16]. Member neurons of such functional assemblies are interpreted to encode the relevant information by synchronizing their spiking activity. Under this assumption the spike synchrony produced by the assembly can be considered as the signal and the remaining stochastic activity as background noise. In order for a downstream neuron to reliably convey and process the incoming signal received from the assembly, it is essential to detect the synchronous input events carrying the signal and to discern them from corrupting noise. Moreover, the processing of such a synchrony-based code must occur independently of the firing rate of the assembly members. We have shown that indeed the presence of afferent spike synchrony leads to increased correlation susceptibility compared to the transmission of shared input correlations. The finding of a correlation susceptibility that is not a function of the firing rate alone [35] demonstrates a limitation of the existing Gaussian white noise theory that fails to explain the qualitatively different correlation transmission due to synchrony.

Though in the limit of weak input correlation the correlation in the output is bounded by that in the input, in agreement with previous reports [37], [35], [58], our results show that for high input correlation caused by synchrony, neurons are able to correlate their outputs stronger than their inputs. This finding extends the prevailing view of correlation propagation as a ‘transmission’, as this notion implies that a certain quantity is transported, and hence can at most be preserved. We have shown in a mechanistic model how this correlation gain results from the non-linearity of cortical neurons enabling them to actively suppress the noise in their input, thus sharpening the signal and improving the signal-to-noise ratio. In convergent-divergent feed forward networks (synfire chains), this mechanism reshapes the incoming spike volley [65], so that synchronized activity travels through the feed forward structure in a stable manner or builds up iteratively from a less correlated state, if the initial correlations exceed a critical value [66], [67]. From our findings we conclude that the boosting of correlation transmission renders input synchrony highly effective compared to shared input in causing closely time-locked output spikes in a task dependent and time modulated manner, as observed in vivo [22].

## Methods

### Impulse Response to Second Order

We here derive an approximation for the integral of the impulse response of the firing rate with respect to a perturbing impulse in the input. A similar derivation has been presented in [12, App. 4.3]. Consider a neuron receiving background spiking input with a first and second moment 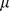 and 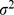, respectively, and an additional incoming impulse of amplitude 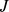 at time 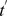. The arrival of the impulse causes an instantaneous shift of the membrane potential by 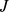. Therefore the probability density at voltage 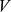 is increased in proportion to the density at 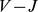 before the jump, whereas the density is decreased by the states that were at 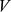. This amounts to an additional term in the Fokker-Planck equation (3), which reads
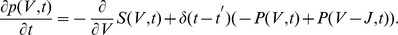
Applying a Kramers-Moyal expansion [41] (a Taylor expansion in 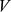 up to second order) to the additional term, we get
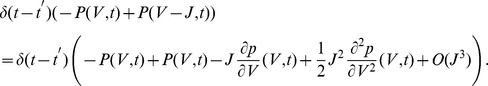
Combining the terms proportional to the first and second order derivative with the corresponding terms appearing in eq:P(V,t) leads to
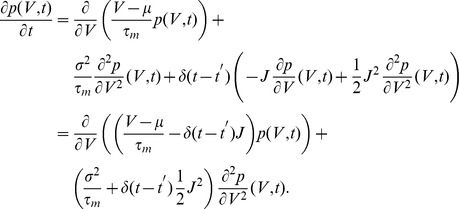
So the additional impulse can be considered as a 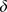-shaped perturbation of the first and second infinitesimal moment. We therefore introduce a formal dependence of 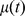 and 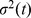 on a time dependent function 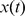 as
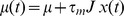


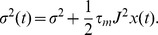
If we are interested in the effect of an impulse of small amplitude 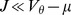, we may linearly approximate the response 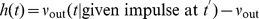 of the neuron to the impulse 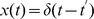. It generally holds that to linear approximation in 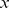 the integral of the response to an impulse 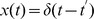 equals the response to a unit-step in the parameter 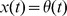, because 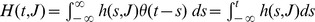. In the limit of 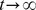 the step response equals the DC-susceptibility, which can be expressed as the derivative with respect to the perturbed quantity 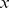. Therefore we obtain to linear approximation
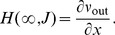
(17)Using the well known expression for the mean first passage time [68], [40] for a neuron with stationary input
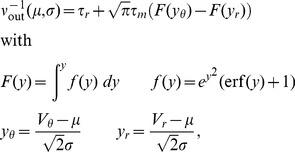
(18)(17) can be evaluated as
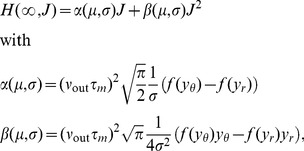
(19)where we applied the chain rule to express 
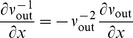
 and 
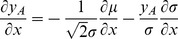
 as well as 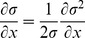, so finally 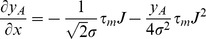 for 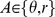.

### Moments of the Binomial Distribution

The first four moments of the binomial distribution 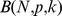 are [69]

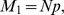


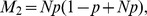


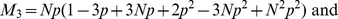


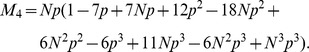



**Table 1:** Parameters of the input and LIF neuron used in the simulations.

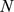	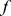	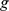	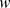	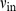	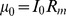	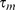	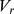	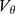	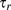
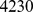	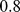	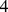	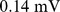	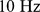	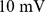	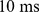	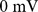	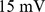	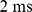
